# Diagnostic and Prognostic Markers in Differentiated Thyroid Cancer

**DOI:** 10.2174/138920211798120826

**Published:** 2011-12

**Authors:** José M Gómez Sáez

**Affiliations:** Endocrinology and Nutrition Service, University Hospital of Bellvitge, L´Hospitalet of Llobregat, Spanish Biomedical Research Centre in Diabetes and Associated Metabolic Disorders (CIBERDEM), Barcelona, Spain

**Keywords:** *AKT*, *BRAF*, *PI3K*, *PTEN*, *RAS*, *RET*, differentiated thyroid cancer.

## Abstract

The MAPK/ERK (mitogen-activated protein kinase/extracellular signal- regulated kinase signaling pathway) and PI3K/Akt (lipid kinase phoshoinositide-3-kinase signaling pathway) play an important role in transmission of cell signals through transduction systems as ligands, transmembrane receptors and cytoplasmic secondary messengers to cell nucleus, where they influence the expression of genes that regulate important cellular processes: cell growth, proliferation and apoptosis. The genes, coding the signaling cascade proteins (*RET*, *RAS*, *BRAF*, PI3K, *PTEN*, *AKT*), are mutated or aberrantly expressed in thyroid cancer derived from follicular thyroid cell. Genetic and epigenetic alternations, concerning MAPK/ERK and PI3K/Akt signaling pathways, contribute to their activation and interaction in consequence of malignant follicular cell transformation. Moreover, it is additionally pointed out that genetic, as well as epigenetic DNA changing *via *aberrant methylation of several tumor suppressor and thyroid-specific genes is associated with tumor aggressiveness, being a jointly responsible mechanism for thyroid tumorigenesis. In the present manuscript the currently developed diagnostic and prognostic genetic/epigenetic markers are presented; the understanding of this molecular mechanism provides access to novel molecular therapeutic strategies.

## INTRODUCTION

More than 10% of the population will develop nodules over their life, and a rise in thyroid cancer has also been observed in the last 3 decades, taking into account the diagnosis of 240,000 new cases in USA in the year 2004 [1]. So, thyroid nodules are very common and with the growing use of diagnostic imaging the number of thyroid nodules identified and undergoing further diagnostic evaluation as fine-needle aspiration biopsy (FNAB) is steadily growing; this technique is a safe, straightforward, sensitive, office-based diagnostic procedure that represents an accepted standard of practice. Nevertheless, only 5% of all thyroid nodules harbor malignancy; therefore, preoperative differentiation of benign from malignant thyroid nodules is imperative. Its main limitation, however, remains the FNAB cytopathological category of indeterminate nodule, as definitive diagnosis of malignancy, requires a morphological finding of capsular and or vascular invasion by the tumor that is only identifiable in a resected thyroid specimen. Thyroid cancer constitutes only 1% of all epithelial malignancies worldwide and it represents 95% of all endocrine malignancies. It is currently unknown whether the increase in papillary thyroid cancer occurrence is real or is a false-positive result of improved diagnostic techniques and other procedures, or of increased screening for small nodules [2,3]. In the aspirate test of nodules after FNAB, 60-70% is considered benign, 5% is considered malignant, and 10-30% is considered uncertain or suspected. Among suspected nodules, 20-25% eventually show thyroid cancer after surgery, and consequently 75-80% of patients in this subgroup will suffer an unnecessary thyroidectomy [4-6]. Although FNAB is the current gold-standard diagnostic test for thyroid nodules, it continues to be limited in the differential diagnosis of indeterminate lesions. In suspected cytolology, one can observe findings of diagnosis superposition among the following: hyperplasic nodules, follicular adenoma, cell adenoma of Hürthle cells, cancer of Hürthle cells and follicular variant of papillary carcinoma. The biology of thyroid cancer ranges from curable, incidental, well-differentiated micro-carcinomas to almost uniformly fatal poorly differentiated carcinomas. Despite extensive research to date, especially in genetic alterations, the scientific community has struggled to translate biomarkers into useful clinical tools and thus attains the full clinical potential for patients presenting indeterminate thyroid nodules. The conclusions following from all this information are that nowadays several markers have been used to improve diagnostic accuracy in the cases of uncertain or suspected cytological tests and mutation detection in clinical FNAB samples from thyroid nodules with the addition of *BRAF* mutation, and also the detection of *RAS, RET/PTC*, and PAX8/PPARγ mutations, may also contribute to cancer diagnosis. *BRAF*, can also be used as a tool for tumor prognostication based in the recent evidences and in clinical practice [7,8]. On the other hand, the MAPK/ERK (mitogen-activated protein kinase/extracellular signal-regulated kinase signaling pathway) and PI3K/Akt (lipid kinase phoshoinositide-3-kinase signaling pathway) play an important role in the transmission of cell signals through transduction systems as ligands, transmembrane receptors and cytoplasmic secondary messengers to cell nucleus, where they influence the expression of genes that regulate important cellular processes: cell growth, proliferation and apoptosis. The genes, coding the signaling cascade proteins (*RET, RAS, BRAF, PI3K, PTEN, AKT*), are mutated or aberrantly expressed in thyroid cancer derived from follicular thyroid cell. Genetic and epigenetic alternations, concerning MAPK/ERK and PI3K/Akt signaling pathways, contribute to their activation and interaction in consequence of malignant follicular cell transformation. Moreover, it is additionally pointed out that genetic, as well as epigenetic DNA changing *via *aberrant methylation of several tumor suppressor and thyroid-specific genes is associated with tumor aggressiveness, being a jointly responsible mechanism for thyroid tumorigenesis. The understanding of this molecular mechanism provides access to novel molecular prognostic and therapeutic strategies for inhibiting oncogenic activity of signaling pathways [7-14]. 

## RET/PTC

Constitutive activation of *RET, RAS*, and *BRAF* which are principle initiators of thyroid cancer. Activation of this tumor initiation pathway occurs in 70% of thyroid cancers. Paracentric reversal with the fusion of the protooncogene *RET* and the gene H4/D10S170 or the gene RFG/ELE1 drives to the active way RET/PTC that codifies a protein with protein kinase activity in 50% of papillary thyroid cancers and also in benign nodules (29.2%), so this activation is not a marker of malignity. 

*RET/PTC* is found in approximately in the 20% of adult sporadic papillary carcinomas, It can be detected in the sample test through inverse and chain reaction transcriptase of quantitative polymerase, which is not usual in clinical practice and is difficult to standardise. Thus, but its prevalence is highly variable between various observations, as consequence of the different sensitivity of the detection methods and also due to geographic variability and genetic heterogeneity [14,15]. All *RET/PTC* chimeric proteins activate the *RAS-RAF*-MAPK cascade and promote tumorigenesis. R*ET/PTC* rearrangement occurs in patients with the history of radiation exposure (50–80%) and also in papillary carcinomas from children to young adults (40–70%) where the PTCs that they presented have different characteristics with the classic papillary architecture, well prognostic and frequent rate of lymph node metastasis. So, the pattern of radiation-induced *RET* and *NTRK1* rearrangements in post-Chernobyl PTC biological has a phenotypic and clinical implications. [15-20]. The distribution of *RET/PTC* rearrangement within the tumor may be also heterogeneous, from clonal *RET/PTC* to be present only in a small fraction of tumor cells, nonclonal *RET/PTC*; this fact difficults also the diagnostic and the characterization of PTC tumors [19-21]. It can be detected in the sample test through inverse and chain reaction transcriptase of quantitative polymerase, which is not usual in clinical practice and is difficult to standardize. In clinical studies, 30-50% of papillary carcinomas are present *RET/PTC* in the cytological sample extracted by FNAB, but its analysis is difficult and may only be useful in combination with other markers [6,10,22]. 

**Fig. (1):**
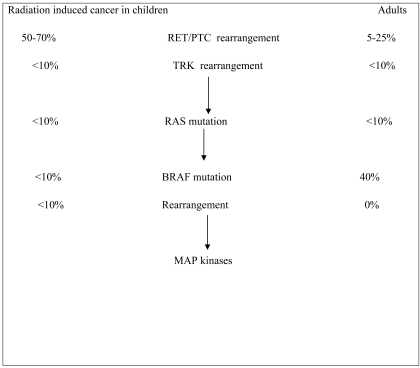
Percentages of genetic alterations found in radiated children or in spontaneous adult papillary thyroid carcinoma.

The rearrangement, *RET/PTC*1 is the most common and comprises up to 60–70% of positive cases, whereas *RET/PTC*3 accounts for 20–30% and *RET/PTC2* and other novel rearrangement types for less than 5% [20]. *RET-PTC* fusions have been reported at different percentage in different studies as well specified by the same author and only subjects (children and young adults) who have been exposed to radiations present a truly high incidence of this particular genetic alteration (Fig. **1**). The *RET/PTC3* was the most frequent after Chernobyl accident [20,21,23].

Correlation between *RET/PTC* rearrangement and prognosis in PTC remains unclear. Some evidence suggests that the *RET/PTC1* rearrangement type is associated with more favourable behaviour of PTC. In contrast to cases presented with *RAS* mutations that are associated with aggressive tumor phenotypes and poor prognosis. PTC cells carrying a *BRAF *mutation are more resistant to sorafenib treatment than PTC cells carrying *RET/PTC* rearrangement. Tumors harboring *RET/PTC*, especially *RET/PTC1*, have a very low probability of progression to poorly differentiated and anaplastic carcinomas [23]. 

## RAS

The activation of *RAS* constitutes a central mechanism in the development of papillary cancer in several cases; in cancer its mutations induce the hyperactivity of thyroid cells, though this phenomenon can also be observed in benign tumors [24-27]. 

Point mutations of the *RAS* genes are not restricted to a particular type of thyroid tumor and are found in follicular carcinomas, papillary carcinomas, and follicular adenomas. But, *RAS* mutations have been shown a trend to be associated to particular thyroid cancer histotypes. Specifically, *N-RAS* with follicular variant PTC, follicular carcinoma and poorly differentiated thyroid carcinomas and perhaps *K*-*RAS* with classical PTC [24]. The genetic alterations in the *RAS/RAF*/mitogen-activated protein kinase and phosphatidylinositol 3-kinase/Akt signaling pathways is present in the follicular variant of papillary thyroid carcinoma [25]. The frequency of *RAS *mutations reported in various series using comparable methodology utilizing DNA sequencing alone to define their role in thyroid tumorigenesis better of all three *RAS *genes (H-*RAS*, K-*RAS*, and N-*RAS*) in the same sporadic (non-radiation-induced) thyroid tumor. Although the frequency of *RAS *point mutation in specific histologic types of benign and malignant thyroid tumors has been widely variable in the literature.* RAS *oncogene activation has been found in nearly 20% of benign and 40% of malignant thyroid tumors. The *RAS *mutations could only be identified in 3% samples with nodular goiter. Mutation of *RAS *gene was not present in the Hürthle cell adenomas. Most *RAS *oncogenes occur in follicular type of tumors and have a higher incidence in follicular carcinomas. However, the reported prevalence of *RAS *mutations in papillary carcinomas was generally low. Several large studies in which tumors of various histologic types were analyzed using a highly sensitive screening method followed by direct sequencing to confirm mutations have also failed to identify any *ras *mutations. The exact causes of the variability in *RAS *mutation in papillary carcinomas remain unclear, they do not appear to harbor mutations of this oncogene in certain population. The significant association between *RAS *mutation and poorly or undifferentiated thyroid carcinomas of follicular cell derivation was confirmed in a recent series in papillary carcinomas [26, 27]. 

The *RAS *proto-oncogene encodes for a 21-kd protein (*Ha*-, *Ki*- and *N-RAS*), which participates in signal transmission from the cell surface to the nucleus. *RAS *activation stimulates cell division and inhibits cell differentiation. The *RAS *oncogene is activated by point mutations in codon 12 or 61 and sometimes in codon 13 or 59. All three *RAS *genes are activated in thyroid cancers at a similar rate (11%–15%). However, *RAS *mutations are rare in solitary benign thyroid nodules. The monoclonal origin of these tumors implies somatic mutations in genes other than *H*a-, *Ki*-, and *N-RAS*. Transgenic mice, in which *RAS *gene expression is specifically targeted to thyroid cells by the thyroglobulin gene promoter, have been reported to develop thyroid hyperplasia, papillary thyroid cancer and follicular adenomas or follicular carcinomas. Reduced expression of differentiation markers has been observed in these tumors, but the mechanism(s) *via* that mutated *RAS *proteins stimulate cell division and inhibit cell differentiation, are poorly characterized. [24,25,28]. The role of *RAS* mutation in predicting more aggressive tumor behaviour is not well defined. Some evidence exists, however, that *RAS* mutations in invasive follicular and papillary carcinoma may correlate with more unfavourable prognosis, but this correlation is far from being conclusively established. 

The study of the alterations and downstream-activated signals of the *RAS/RAF*-mitogen-activated protein kinase (MAPK) and phosphatidylinositol 3-kinase/v-akt murine thymoma viral oncogene (*Akt*) (PI3K/Akt) signaling pathways. Tumors and matched normal thyroid samples were tested for *RAS*, for the v-*RAF* murine sarcoma viral oncogene (BRAF) substitution of valine (V) for glutamate (E) at codon 600 (the V600E mutation), for phosphatase and tensin homolog (PTEN), for catalytic phosphatidylinositol 3-kinase p110 subunit alpha (PIK3CA), for *AKT*, and for the presence of rearranged during transfection (*RET*) proto-oncogene/PTC (*RET-PTC*) and paired box-8 (PAX8)/peroxisome proliferator-activated receptor gamma (PPARã) fusion protein (PAX8-PPARγ) rearrangements by direct sequencing and reverse transcriptase-polymerases chain reaction analyses, respectively. Genetic alterations have been identified in 70% of follicular variant of PTCs. Activation of the MAPK and phosphatidylinositol 3-kinase pathways was observed in 74% and 22% of tumors, respectively. The alterations that were identified in the genes of the 2 pathways were mutually exclusive. Chromosomal *RET-PTC* and PAX8-PPARγ rearrangements were observed in 20% and 17% of tumors, respectively. Thus, *RET-PTC* and PAX8-PPARγ rearrangements and mutations of the neuroblastoma *RAS* viral oncogene homolog N-*RAS* at codon 61 were the most common genetic alterations in follicular variant of PTCs. Activation of the MAPK pathway was a frequent event in follicular variant PTCs, and the phosphatidylinositol 3-kinase signaling pathway could be coactivated in *RET-PTC* tumors. These findings may have important therapeutic implication in patients with follicular variant of PTC [25]. Information obtained through cytological smears or anatomic samples permits the study of complex metabolic pathways, thus providing researchers with a high throughput tool for elucidating changes in the global expression patterns seen in tumor cells. This ability to investigate tumor biology would allow the selection of different drugs, considering the predominant altered pathways observed in these samples [29,30]. a. Compounds *RAS* antisense. These are small complementary DNA sequences with a specific target of the mRNA that would be useful as a substratum for ribonuclease to interfere in ribosomes, block the expression of genes, and inhibit the synthesis of protein. There are 2 drugs in development, ISIS 2503 and ISIS 5132 [29]. b. Fenilacetat. This comes from the metabolism of phenylalanine and affects the translational process of *RAS*; it also decreases tumor growth and cellular differentiation, increases the 131I uptake and synthesis of thyroglobulin, and interferes in angiogenesis. In follicular cancer, fenilacetat would also act in synergy with transretinoic acid [29,30]. c. Farnesil transferase inhibition. It has been proved that this inhibits the accumulation in *in vitro* of *RAS*, thus reducing its signal of transduction. d. *RAF* inhibition. The *RAF* phosphorilated active MAP kinase promotes cell growth and decreases cell death; there is a compound *RAF* antisense that is able to inhibit tumoral growth as much in *in vitro* as in *in vivo* [30]. 

## BRAF 

*BRAF* is a central regulator of thyroid-specific differentiation and proliferative capacity in *in vitro* benign thyroid cell models. *BRAF* belongs to the RAF family of serine/threonine kinases and the V600E mutation results in constitutive activation in 45% of papillary thyroid carcinomas in adults. *BRAF* V600E mutation is typically found in papillary carcinomas with classical histology and in the tall cell variant, and is less common in the follicular variant of papillary carcinoma. This mutation can also be seen in anaplastic and poorly differentiated thyroid carcinomas arising from papillary carcinoma [31-34].

*BRAF* mutations could occur in other exons of the gene. However the probability for this assumption is rather low This signaling cascade is not necessary in the initiation of tumor growth in thyroid follicular cells. This corresponds to a recent *in vitro *finding that suggests that the dedifferentiated phenotype of cold thyroid nodules is unlikely to be the result of activated *RAS *signaling. About 95% of all mutations involve nucleotide 1799 and result in a substitution of valine to glutamate at residue 600 (V600E). This point mutation leads to constitutive activation of BRAF kinase and chronic stimulation of the MAPK pathway, and is tumorigenic for thyroid cells About 95% of all mutations involve nucleotide 1799 and result in a substitution of valine to glutamate at residue 600 (V600E). This point mutation leads to constitutive activation of BRAF kinase and chronic stimulation of the MAPK pathway, and is tumorigenic for thyroid cells [31,32]. Other and rare mechanisms of *BRAF* activation in thyroid papillary cancer include K601E point mutation, small in-frame insertions or deletions surrounding codon 600.

Moreover mice with transgenic expression of activated *RAS *targeted to the thyroid show thyroid abnormalities only with low incidence after long latency and develop papillary carcinomas rather than follicular tumors. Together with the recent finding of *BRAF* mutations in papillary thyroid carcinomas activation of *RAS *or downstream signalling might not be a strong requisite for tumor progression and malignant transformation of follicular thyroid lesion. There are molecular events other than *BRAF* or *RAS* mutations that could constitutively activate the *RAS/RAF/MEK/ ERK/MAP* pathway. Such candidate molecules include other members of the *RAF* gene family such as *RAF*-1 or downstream genes such as ERG [33]. However, mutations in these genes have not been reported in neoplasia so far. 

Detection and molecular characterization of a novel *BRAF* activated domain mutation in follicular variant of PTC; this finding reflects the importance to detect, in addition to the V600E mutation, other important activating *BRAF* mutations at least in follicular variant of papillar thyroid carcinoma [33]. The *BRAF* V600E mutation represents more than 90% of *BRAF* mutations found in thyroid cancer [34]. Based on evidence that *BRAF* is involved in the development of papillary carcinoma and in the progression to anaplastic cancer, *BRAF* is an attractive target in thyroid cancer, especially anaplastic and aggressive papillary subtypes where there is an urgent need for treatment. *BRAF* V600E can induce thyroid cell transformation in *in vitro* and thyroid cancer in *in vivo*, confirming that this mutation is an oncogene for thyroid cancer [9,10]. *BRAF* V600E mutation is reported in approximately only 25% of anaplastic thyroid cancer suggesting that other genetic markers contribute to tumor progression. Studies that reported higher percentage of *BRAF* mutations in anaplastic thyroid cancer used poor sensitive detection technologies [31,35,36]. 

The application of *BRAF/*V600E mutation analysis in FNAB specimens is more effective for thyroid nodules with malignant echographic features as compared with nodules without malignant echographic features. The use of the AS-PCR is more valuable as compared with the direct DNA sequencing to refine the diagnosis in a clinical setting [37]. 

These findings might have some theoretical *BRAF* V600E mutation is generally accepted as a reliable prognostic marker for papillary carcinoma and more importantly, has been found to be an independent predictor of treatment failure and tumor recurrence, even in patients with low stage disease [38-41]. 

*BRAF* activation *via BRAF* V600E mutation in thyroid cells appears to lead to the alteration of function of sodium iodide symporter (NIS) and other genes metabolizing iodide, which is likely to be responsible for the decreased ability of tumors with *BRAF *mutation to trap radioiodine and treatment failure of the recurrent disease [41-43]. The prognostic implications of *BRAF* mutation should be of particular importance in papillary microcarcinomas, which are incidentally discovered tumors. *BRAF *V600E is the leading genetic event for PTC formation and the prevalence of *BRAF* mutation in clinically evident PTC in comparison to a set of and preoperatively discovered microcarcinomas the difference in *BRAF *V600E prevalence was in clinically evident PTC was 45% and in microcarcinomas 38.3%. It has been demonstrated that *BRAF* mutation in thyroid microcarcinomas correlates with either high rate of extrathyroidal tumor extension or lymph node metastasis [43]. In this sense, the combinational use of the mutation markers discussed above might be prognostically useful since *RAS* mutation, *RET/PTC*, and likely PAX8/PPARγ predict a less aggressive course of thyroid cancer than the *BRAF* mutation. Therefore, additional studies are needed to identify the highest risk patients within the BRAF mutation–positive group [44-47].

The incidence of *BRAF* V600E in anaplastic carcinomas is similar to that in early-stage well-differentiated tumors, suggesting that some anaplastic carcinomas develop from PTC and that *BRAF* signaling may be important in this process [47]. In human thyroid cancer, *BRAF* V600E is associated with vascular endothelial growth factor (VEGF) over-expression, which in turn is associated with increasing tumor stage and invasiveness [45-48]. *BRAF* V600E as a target may not be limited to only PTC with a *BRAF* activating mutation, since *BRAF* and other RAF kinases are activated by other oncogenes involved in follicular and papillary carcinoma [7]. Extensive angiogenesis in thyroid cancer cells has been observed. Thyroid tumors are highly vascular and follicular cancers that metastasize through blood vessels. Cancers expressed more VEGF mRNA and protein than did normal thyroid tissue. The mRNA expression patterns and immunohistochemical staining were similar between primary thyroid tumors and their lung or lymph node metastases. VEGF expression and secretion was increased in the differentiated thyroid cancers, follicular and papillary [46,48]. RAF kinases set off a mitogenic cascade of events that modulate gene expression by phosphorylation of transcription factors, which in turn affect cell proliferation and malignant transformation. There are 3 members of the RAF kinase family: *ARAF*, *BRAF* and *RAF*-1. Additional evidence suggests that *BRAF* and *RAF*-1 take part in regulation of endothelial apoptosis and angiogenesis [49]. Sorafenib has demonstrated ability to inhibit *RAF*-1 and tumor cell line proliferation as well as tumor growth in multiple human tumor xenograft models. It was shown that sorafenib inhibits wild type (WT) *BRAF* and V600E mutant *BRAF*, as well as VEGFR-2, platelet-derived growth factor receptor (PDGFR)-β and VEGFR-3. In this same study, sorafenib exhibited antitumor activity in xenograft models of human breast, colon, ovarian, lung, melanoma and pancreatic cancers, demonstrating effectiveness against tumors with mutations in *KRAS* and *BRAF* [50]. Sorafenib treatment also resulted in a 50 to 80% inhibition of microvessel area and density in a colon xenograft harboring mutated BRAF. These data suggested that sorafenib is both a RAF kinase and VEGFR inhibitor that targets the *RAF/MEK/ERK* cellular proliferation pathway and the receptor tyrosine kinases that support tumor angiogenesis [51]. *RET* is constitutively active in medullary and PTC. Medullary carcinoma has often metastasized before diagnosis, rendering it incurable. If initial measures for papillary carcinoma fail, these subjects too will be unable to be cured. For these subjects, there is no currently effective treatment. Mutations in *RET* in medullary, papillary and familial thyroid carcinoma make *RET* another promising target for the treatment of thyroid cancer [52,53]. Some authors showed that sorafenib inhibits the enzymatic function of the *RET-PTC* fusion protein and *RET* signaling, including receptor autophosphorylation and downstream signaling in a panel of human thyroid cancer cells with activating *RET* mutations along with *RET/PTC* and *RET*/multiple endocrine neoplasia (MEN) 2 oncogenes [54]. Sorafenib also targets signal transduction along the MAPK pathway and proliferation of tumor cells in *BRAF* V600E-positive thyroid cancer cell lines. Studies have shown that sorafenib can inhibit proliferation of poorly differentiated thyroid cancer cell lines regardless of whether they harbor *BRAF* V600E mutations [55]. Nowadays, the main criteria is that sorafenib mainly affects through inhibition of angiogenesis rather than specifically inhibiting the *RAS* pathway. Several phase II studies have proved this concept (e.g. melanoma and renal cancers phase clinical trials) [52,53]. Activating *BRAF* mutations in papillary thyroid carcinomas have been linked to aberrant methylation of several tumor-suppressor genes, including *TIMP3*, *SLC*5A8, *DAPK* and *RAR*β2 [56,57]. Methylation of these genes correlated with signs of aggressive behavior in thyroid neoplasms, including extrathyroidal invasion, lymph node metastasis and advanced tumor stage at diagnosis, and their epigenetic silencing may be an important mechanism by which *BRAF* mutation promotes cancer progression [58,59]. Tyrosine kinase receptors bind for a wide variety of ligands and frequently are mutated and induce a constitutive activation such that a chimerical protein expression takes place in follicular cells in the domain of *RET*, as well as in other receptors. a. Receptors of vascular endothelial growth factor (VEGF). There are three receptors, 1,2 and 3, and it seems that 2 would prevail in the transduction pathway. There are studies that show high concentrations of VEGF in the serum of patients with metastases [7-10]. The drugs vandetanib, sorafenid, motesanib, sunitinib and exelisis are directed against *RET*; sorafenid and vandetanib against *BRAF*, and against VEGFR: axitinib, vandetanib, sorafenid and motesanib. b. Epidermal growth factor receptors (EGF). These constitute a superfamily of 4 related receptors [51], of which the most relevant is the Her2/neu that is over-expressed in thyroid cancer. Taking into account the relevance of the 2 receptors VEGF and EGF, attention is now being dedicated to the possible blockade of the same in different levels of the inhibition of their m RNA with antisense compounds [51]. c. Antibodies. Monoclonal antibodies have been designed against the VEGF, and are able to reduce angiogenesis; some of them are in phase II or III of development. In experiments on animals, it has been proved that anti-VEGF antibodies block the angiogenesis and the expression of p53. Additionally, monoclonal antibodies have been developed against EGF, whose clinical studies in combination with chemotherapy or radiotherapy, are being given information about their possibilities. Herceptine is a high- affinity monoclonal antibody against the Her2/neu expressed by cancer, but herceptine not has been assayed in thyroid cancer [52]. d. Antagonists of the receptors. VEGF receptor inhibition through a selective antagonist of the receptor 2 is being used in studies in combination with placlitaxel in advanced cases. Paclitaxel in combination with campothectin has also been used because of its cytotoxic action in the cellular medullary carcinoma lines of the human thyroid [52]. e. Small molecules. These are small molecules that can interfere with receptors, with promising results, because they can block the tyrosine kinase activity of EGF. One of these molecules, ZD6474, manages by the oral route to inhibit angiogenesis through the inhibition of VEGF, but it has even been proved that it would inhibit the family of *RET* oncoproteins as much in *in vitro* as in *in vivo*, including *RET*/MEN2A, the *RET*/MEN2B and the *RET*/*PTC3*. This would suggest the potential of ZD6474 in the treatment of both medullary and papillary carcinoma [52, 58]. Treatment directed against tumoral angiogenesis. 1. Thalidomide. Designed initially as a sedative, it has been proved to possess antineoplasic properties through its antiangiogenic actions of unknown origin. At present, a study gives information in a phase II assay. 2. Combretastatines. These are a protein family that is united to tubulin with antiangiogenic and antineoplasic properties; a study has been completed with combretastatine A4 in anaplastic carcinomas in which complete remission was observed in a patient [53, 59]. 

## PAX8/PEROXISOME-PROLIFERATOR-ACTIVATED RECEPTORγ FUSION

PAX8/peroxisome-proliferator-activated receptorγ fusion (PPARγ) rearrangement is a ligand-dependent transcription factor and member of the nuclear receptor superfamily. It gained first attention as a key regulator of adipocyte differentiation, a process paralleled by cell cycle arrest and has been identified as a target for thiazolidinediones used as insulin sensitizers in the treatment of diabetes mellitus. A more general role of PPARγ in regulation of cell differentiation and proliferation has been proposed on the basis that PPAR*γ *agonists thiazolidinediones may prevent formation of neoplastic lesions in animal tumor models, exert growth-inhibitory effects in addition to reinduction of differentiation in several human cancers and in tumors induce changes in the cell cycle with growth arrest and apoptosis. PPARγ has also been linked with thyroid pathology since the discovery of a PAX8/PPARγ rearrangement in follicular thyroid carcinomas. This rearrangement, which represents a t(2;3)(q13;p25) translocation involving PAX8, an important transcription factor for thyroid differentiation and PPARγ results in a fusion gene able to suppress wild-type PPARγ in a dominant negative behaviour. PPARγ has gained attention in various cancers, where impairment of PPARγ function through mutations has been observed. Although little is known about the precise molecular mechanism of PAX-8/PPARγ action, it is reasonable to assume that competition in transcriptional activation through DNA binding between overexpressed PPARγ-fusion gene and wild-type PPARγ could be the likely mechanism of dominant-negative PPARγ inhibition. 

The demonstration of the PAX8/PPARγ fusion oncogene in a subset of follicular thyroid tumors provides a new and promising starting point to dissect the molecular genetic events involved in the development of this tumor form [60]. There are recent studies that prove the importance of genetic translocation between regions 3p25 and 2q13 when that translocation supposes the fusion of transcription thyroid factor PAX8 with the PPARγ that is an oncogene. This fusion element increases cell growth, reduces apoptosis, and blocks the union of cellular lines to the surrounding tissues. It has been described mainly in follicular carcinoma, but is not a specific marker of this thyroid cancer [60]. 

PAX8/PPARγ is found in 30–40% of conventional-type follicular carcinomas, and with lower prevalence in oncocytic carcinomas in a small fraction of follicular adenomas and occasionally in the follicular variant of papillary carcinoma. Tumors harboring PAX8/PPARγ tend to present at a younger age and are smaller in size, have a solid/nested growth pattern, and more frequently reveal vascular invasion [7, 61]. 

### MicroRNA markers

(miRNAs) are are a newly discovered class of endogenous short (18–24 nucleotides) noncoding RNAs. that act as negative regulators of the protein-coding gene expression through the complementary binding to 30 untranslated region (UTR) of target mRNA, which lead to translational repression and inhibition of protein synthesis. miRNA expression is deregulated in many types of human cancers, including thyroid cancer [62,63]. Several studies have demonstrated that normal thyroid cells have a unique profile of miRNA expression and many miRNAs are dysregulated in thyroid cancer cells. A subset of these miRNAs, including miR-221, miR-222, miR- 146b, miR- 155, and miR- 187, has been consistently found to be upregulated in thyroid papillary carcinoma. As the most important innate immune receptors, toll-like receptors (TLRs) represent the first line of defense against pathogens. MiRNAs have emerged as important controllers of TLR signaling. Several miRNAs, induced by TLR activation in innate immune cells, target the 3' untranslated regions of mRNAs encoding components of the TLR signaling system. miRNAs also function as an important link between the innate and adaptive immune systems, and their dysregulation may have a role in the pathogenesis of inflammatory diseases [64]. 

They are *BRAF* and *RAS* point mutations and *RET/PTC* and PAX8/PPARγ rearrangements. Among other types of genetic alteration, miRNA markers appear to be the most promising for the diagnostic use at this time miRNAs [62].

As many as 530 human miRNAs have been identified and this number is constantly increasing. miRNAs have a role in a wide variety of physiologic cellular processes, including differentiation, proliferation, and apoptosis. miRNA dysregulation is a common finding in malignancy, and there is mounting evidence supporting a role of miRNAs in carcinogenesis [62].

Specific tumors exhibit unique expression profiles of miRNAs differentiating them from one another, more importantly from normal tissues. Several recent studies have utilized miRNA microarrays to demonstrate unique molecular expression signatures differentiating benign from malignant thyroid lesions, particularly PTC, which is the most common well-differentiated thyroid malignancy encountered in clinical practice. Four mutation types constitute the majority of known mutations occurring in papillary and follicular cancers and carry the highest impact on tumor diagnosis and prognostication. Specifically, miRNAs 21, 31, 146b, 187, 221, and 222 are differentially expressed in malignant and normal thyroid tissue [64]. Moreover, strong correlation was found between miRNA expression and somatic mutations found in this tumor type. Specifically, upregulation of miR-187, miR-146b, and miR-155 was found to be significantly more pronounced in papillary carcinomas carrying *RET/PTC* rearrangements, a genetic event characteristically found in radiation-induced thyroid tumors.

Many miRNAs are expressed in cell type–specific manner and significantly overexpressed or down-regulated in tumors as compared to normal tissues, providing a rationale for their potential diagnostic use [65,66]. To date, FNAB accuracy is based solely on cytohistological characterization of the aspirated cells and is highly dependent on the expertise of the examiner. Follicular lesions are a subgroup of possible FNAB results that faces both the patient and the treating physician with a dilemma. On one hand, malignancy cannot be definitely diagnosed by FNAB; on the other hand, the actual final malignant pathology rate ranges between 20% and 37%. An accurate diagnostic miRNA panel can bring a dramatic change in the decision process of such cases. miRNA quantification for differential diagnosis of thyroid neoplasms within aspiration biopsy samples is feasible and may improve the accuracy of FNAB cytology [65-70]. 

Deregulated miRNAs have been associated to genetic alterations; to specify also which specific type of mutations or rearrangements have been associated to. It would stress the fact that despite few miRNAs have been associated to some genetic mutations that additional and larger studies are warranted; for example there are few studies that do not find any association between *BRAF* mutations and any miRNAs [65]. One hundred percent specificity and 95% sensitivity were achieved by testing mir-221, and this may reduce the value of the other miRNAs in the panel. However, one must realize that thyroid neoplasms are variable and may represent a common phenotype of many genetic abnormalities. In larger cohorts of patients, these underlying genetic abnormalities may have an impact on miRNA expression [69,70]. At present, the exact role of miRNAs in carcinogenesis of tumors is yet to be elucidated. miRNAs regulate gene expression at the post-transcriptional level and thereby may control cellular processes such as developmental transitions, organ morphology, cell proliferation, and apoptosis. It is postulated that each miRNA regulates up to 100 different mRNAs and that more than10.000 mRNAs appear to be directly regulated by miRNAs [7]. In carcinogenesis, miRNAs can either regulate known oncogenes or tumor suppressor genes at the post-transcriptional level or act themselves as oncogenes or tumor suppressor genes [66]. If up-regulation of these miRNA was found in thyroid cells after radiation exposure, it would suggest that miRNAs play a direct role in the generation of carcinogenic chromosomal rearrangements in human cells. However, none of these miRNAs were found to be up-regulated either after 4 or 24 hours after exposure. This indicates that miRNAs are unlikely to participate directly in the generation of carcinogenic chromosomal rearrangements after radiation exposure, but more likely to play a role in the cellular response to radiation by modulating the expression of acute exposure of thyroid cells to γ-radiation results in several specific patterns of miRNA response and it is likely to affect other cell functions, such as DNA repair [68]. 

### Protein p53

Protein p53 is a codified phosphoprotein and a suppressor gene that is united to specific sequences of DNA regulating cell cycles, inhibiting cell division, repairing DNA and promoting apoptosis. Mutated forms are dominant and their half life is of hours instead of minutes which is the case with non-mutant forms; this allows protein p53 detection by immunochemistry to be relatively easy. About 50% of all cancers present mutations of protein p53, which thus constitute the most frequent genetic alteration in general cancer. Such mutations are observed in between 0-10% of papillary thyroid cancers, in 40% of only-slightly differentiated cancers, and in between 60-90% of anaplastic carcinomas. The presence of mutant protein p53 has been related to a worse prognostic of prognosis for thyroid cancers, but nowadays it is not thought that protein p53 may trigger the transformation of nodules from benign to malignant. This protein therefore has great potential as an indicator of prediction in little-differentiated cases or in anaplastic carcinomas [4,69,70], but not in the aspiration biopsy sample taken from it, and this suggests a sampling error, an inherent limitation of aspiration biopsy. Therefore, theoretically our assay may reach 100% accuracy if sampling accuracy is verified [4,71]. Many thyroid cancers have lost suppressor gene p53 expression because its mutation renders the gene inactive. The restoration of the native p53 way has been used in different experimental models and even in clinical assays. Some studies have proved that the restoration of the gene p53 protects transducted cells as well as the surrounding cells. Its effect is maintained through its antiangiogenic action, since it regulates, VEGF decreasing its activity and increasing trombospondin, which is a strong angiogenenic suppressant. Two recombinant adenoviruses have been used (Cre/loxP system): one contains the promoter expression of p53, and the other, two units. One of these is the gene E1A and the other is a low gene of cytomegalovirus promoter. The coinfection by the two adenoviruses leads to the arrest of viral replication if p53 is present [70,71]. The protein family Gadd45 (growth arrest and DNA damage-inducible gene family), has been implicated in DNA replication and repair; a low concentration of Gadd45 has been shown in anaplasic carcinomas, as has the fact that the increase of Gadd45 mediated by adenoviruses significantly inhibits cellular growth [71]. High mobility group proteins (HMGI) are overexpressed in several malignant tumors; it has been proved that using adenoviruses that transport HMGI with an antisense orientation induces an apoptosis increase in the human cellular lines of anaplastic carcinoma, although not in human cells. Given such a generalised expression of these proteins in thyroid cancer, this strategy could be useful in the future [71]. 

## GALECTIN-3

Galectin-3, formerly known as L-14, gal3 macrophage galactose-specific lectin or HLBP14, is part of a family of lectines not integrins, that transports β-galactoside, regulates growth, has cytostatic functions, transports lamininin, and intervenes in molecular adhesion, inflammation, the malignant transformation of nodules, and the development of metastases. It can be determined in the smear test through immunochemistry and western blotting. Galectin is expressed in thyroid cancer, and its distribution is cytoplasmtic and nuclear [72]. It was first detected in papillary carcinoma, and later in follicular carcinoma, as well as in medullary carcinoma [72,73]. It can be determined in cytogical samples or in surgical pieces through the commercial antibody for immunohystochemical method; it is overexpressed in the majority of follicular carcinomas and also in adenomas, between 1-72%, according to the accumulated results of 8 clinical series with several cases [74-76]. For this reason, it is thought that galectin-3 does not reliably distinguish adenomas from carcinomas. Furthermore, galectin-3 might be able to represent a function in benign to malign nodule transformation [4]. The diagnostic accuracy of the positivity of galectin-3 for inmunohystochemistry presents a sensitivity of 86%, a specificity of 36%, a predictive positive value of 53%, and a predictive negative value of 75%; moreover, it has a diagnostic accuracy of 59%. It can be used, therefore, as a supplementary marker, and galectin-3 could be a good tool for guiding therapeutic decisions in patients with thyroid nodules and with FNAB results suggestive of follicular neoplasm. Nonetheless, the information available shows that nowadays galectin-3 has methodological flaws that preclude a definitive answer about its utility in the clinical setting. Serum concentrations of galectin-1 and -3 are relatively high in patients with thyroid malignancy, but there is a considerable overlap in serum galectin-3 concentrations among those patients with benign and malignant nodular thyroid disease and, to a lesser extent, among those with or without nodular thyroid disease [76]. The combined detection of galectin-3 and *BRAF *V600E improves the diagnosis in FNAB with cytological findings that give rise to suspicions of papillary thyroid carcinoma; and this detection finds clinical application in selected cases. With the combined use of HBME-1 and galectin-3 in indeterminate FNABs, a 10% increase in sensitivity is achieved. These markers show excellent sensitivity and specificity and may improve the selection of patients for surgery [75,77]. 

## EPIGENETIC CHANGES IN THYROID CANCER 

An increasing body of evidence suggests that epigenetic changes (DNA methylation, remodeling and post-translational modification of chromatin) play important roles in thyroid tumorigenesis, as a result of their effects on tumor-cell differentiation and proliferation. Epigenetic silencing of various thyroid-specific genes has been detected in thyroid tumors. These changes can diminish the tumor's ability to concentrate radioiodine, which dramatically reduces treatment options. Epigenetic changes in tumor-promoting and tumor-suppressor genes also contribute to the dysregulation of thyrocyte growth and other aspects of tumorigenesis, such as apoptosis, motility and invasiveness [57,78]. Progress in the field of thyroid cancer genetics has produced a novel class of drugs known as ‘targeted therapeutics’, which act selectively on cancer cells harboring particular genetic aberrations [78], and these agents are undergoing clinical testing for the treatment of aggressive thyroid carcinomas [79]. The differentiation and proliferation properties of thyroid cancer cells are also strongly influenced by epigenetic alterations [80], which are thought to be equally, if not more, important than mutational events in the generation and progression of human cancer [81]. Encouraging preliminary results that have been obtained with epigenetic treatment strategies in several forms of cancer and knowledge of the epigenetic changes that occur in thyroid carcinomas (often in combination with genetic alterations) is expected to reveal more effective ways to treat tumors of this type that fail to respond to currently available treatment modalities [81-85]. The cloning of the NIS gene has supposed the development of a cytoreducting gene therapy based on the transfer of the gene followed by the administration of 131I. Recently, the stable transfection of the NIS gene over cells of follicular carcinoma (FTC-133) without NIS has been achieved, restoring the skills of 131I uptake [86-89]. Additionally, in several tumors NIS gene transfection has been proposed together with a transfection of thyroid peroxidase that might be able to increase the 131I uptake in tissue, as well as the amount of irradiation [90]. Preclinical studies have furnished convincing evidence that deacetylation inhibitors and demethylating agents are beneficial in the treatment of thyroid cancer, and these drugs are now being tested against metastatic radioiodine-refractory thyroid carcinomas. Despite promising phase-I results, however, histone deacetylases treatment of 16 patients with differentiated thyroid carcinoma produced no partial or complete responses that met response evaluation criteria in solid tumors [79] and in a phase-II study, i.v. injection of depsipeptide restored radioiodine avidity in 2 of the 20 patients treated, but there were no objective responses even after 131I treatment [79]. In light of these preliminary data, epigenetic strategies seem far less promising than approaches that target protein kinases. Indeed, protein kinase antagonists have produced decidedly better response rates in clinical trials (although they are by no means free of adverse effects); however, the safety and efficacy of other HDACi and demethylating agents, alone or combined, are still being assessed. Until the results of these trials become available, research on epigenetic alterations in thyroid cancer must continue with the ultimate objective of developing more effective treatments for these tumors [81-84]. 

## OTHER GENETIC ALTERATIONS 

### Telomerase

Telomeres are repetitive sequences of DNA that contribute to its stability, decrease with every cell division, and lead to apoptosis. Telomerase is an enzyme with a protein and an RNA component that adds repetitions at the end of DNA and is not usually present in adult tissues. It has been proved that many tumors present telomerase activity that immortalizes neoplasic cells. Such cells are determined by a polymerase chain reaction and enzyme-linked-immunoabsorbent assay or by telomere repeat-amplification protocol. Telomerase is simply an auxiliary instrument in the differential diagnosis of thyroid cancer [90, 91]. 

### Type I Receptor for Transferrin (TfR1/CD71)

Type I receptor for transferrin (TfR1/CD71) is overexpressed in several malignant tumors, but no studies are available on thyroid carcinomas. Our previous comparative analyses of the relative distribution of transferrin in benign versus papillary thyroid carcinoma tissues highlighted a marked malignancy- associated abundance of the molecule. Some results suggest that altered expression of TfR1/CD71 may be used as a marker helpful in distinguishing papillary thyroid carcinoma from papillary hyperplasia and follicular variant from benign follicular-patterned lesions. Additionally, the present observations support the rationale for the use of radiolabeled transferrin/transferrin analogs and/or anti-TfR1/CD71 antibodies for diagnostic and/or radiotherapeutic purposes in TfR1/CD71-expressing thyroid tumors [92]. 

A great number of those that are implicated in carcinogenesis or that are expressed in tumors of different ways have been used. They have multidisciplinary characteristics and are of difficult interpretation given their complexity and the variety of results that have been obtained [93, 94]. Thus, a growth inhibitor of the signal of transduction has been used as the caveolin that intervenes in the cellular cycle and also 14-3-3 α, a regulator of the cellular cycle and the cyclines and kinases dependent of the cyclines as well as the non-dependent [95,96]. As a marker, S100A9, a binding protein of calcium related to the cell differentiation that would be a marker of dedifferentiation has also been studied [8]. Different adhesion molecules have also been analysed, since these are found overexpressed in carcinomas. Furthermore, markers of natural lymphocyte killers have been studied, such markers such as CD57, a lymphocytes natural killer, whose increased expression would be present in cancers [97], as well as that of the glucose transporter (GLUT-1) [98]. Other markers such as epidermal growth factor receptors, oncoproteins with growth capacity and tyrosine kinase activity have been analysed, but the results are difficult to interpret and are controversial in some cases [99]. Several genes are candidates for the tumorigenesis of thyroid cancer and have also been analysed, especially those resulting from the microprobe development that allows the analysis of multiple genes and from the global pattern selection that elucidates which of these genes appear in tumor cells; this field is nowadays one that raises a great degree of expectation [100]. The recent discovery of anaplastic lymphoma kinase (*ALK*) gene mutations in thyroid cancer may rationalize clinical evaluation of ALK inhibitors in this setting. In undifferentiated anaplastic thyroid cancer, two novel point mutations, C3592T and G3602A, found in exon 23 of the *ALK* gene, with a prevalence of 11.11%, but found no mutations in the matched normal tissues or in well-differentiated thyroid cancers. These two mutations, resulting in L1198F and G1201E amino acid changes, respectively, both reside within the ALK tyrosine kinase domain where they dramatically increased tyrosine kinase activities. Similarly, these mutations heightened the ability of *ALK* to activate the phosphatidylinositol 3-kinase (PI3K)/Akt and mitogen- activated protein (MAP) kinase pathways in established mouse cells. Similar oncogenic properties were observed in the neuroblastoma-associated *ALK* mutants K1062M and F1174L but not in wild-type *ALK*. These findings reveal two novel gain of function mutations of *ALK* in certain, undifferentiated anaplastic thyroid cancer and they suggest efforts to clinically evaluate the use of *ALK* kinase inhibitors to treat patients who harbor undifferentiated cancers with these mutations [101]. The role of the phosphatidylinositol-3 kinase regulatory subunits in differentiated and undifferentiated thyroid carcinomas also in amplifications and mutations is also important and the activation of this pathway is a contribution in thyroid cancer progression; clear understanding of the role played by components of this signaling pathway could be important for the understanding of thyroid tumor progression and the development of novel compounds to be used in clinical trials. The role of β-catenin and phosphatidylinositol-3 kinase regulatory subunits is important either it is in differentiated or in undifferentiated thyroid carcinomas [102,103]. Proteomic has been introduced recently, though not for the systematic study of the samples extracted by FNAB, but its results serve to validate different potential markers, confirming in the current studies that the best of these would be galectin-3, galectin-1 and the protein S100C48 [104]. Regarding specific mutations that constituted the panel for thyroid cancer diagnostic, *RAS*, *BRAF*, *RET/PTC*, and PAX8/PPARγ mutations had all a 100% positive predictive value for cancer. Patients with these mutations would be candidates for total thyroidectomy irrespective of the cytologic diagnosis [43].
